# Exploring the Use of Sensorial LTP/LTD-Like Stimulation to Modulate Human Performance for Complex Visual Stimuli

**DOI:** 10.1371/journal.pone.0158312

**Published:** 2016-06-24

**Authors:** Felipe Pegado, Hendrik Vankrunkelsven, Jean Steyaert, Bart Boets, Hans Op de Beeck

**Affiliations:** 1 Department of Brain and Cognition, KU Leuven, 3000 Leuven, Belgium; 2 Department of Neuroscience, Child & Adolescent Psychiatry, KU Leuven, 3000 Leuven, Belgium; 3 Research group Leuven Autism Research, KU Leuven, Leuven, Belgium; Ecole Polytechnique Federale de Lausanne, SWITZERLAND

## Abstract

Is it possible to passively induce visual learning/unlearning in humans for complex stimuli such as faces? We addressed this question in a series of behavioral studies using passive visual stimulation (flickering of faces at specific temporal frequencies) inspired by well-known synaptic mechanisms of learning: long-term potentiation (LTP) vs long-term depression (LTD). We administered a face identity change detection task before and after a passive stimulation protocol to test for potential changes in visual performance. First, with bilateral stimulation, subjects undergoing high-frequency LTP-like stimulation outperformed those submitted to low-frequency LTD-like stimulation despite equivalent baseline performance (exp. 1). Second, unilateral stimulation replicated the differential modulation of performance, but in a hemifield-specific way (exp. 2). Third, for both stimulation groups, a sudden temporary drop in performance on the stimulated side immediately after the stimulation, followed by progressive recovering, can suggest either ‘visual fatigue’ or ‘face adaptation’ effects due to the stimulation. Fourth, we tested the life-time of these modulatory effects, revealing they vanish after one hour delay (exp. 3). Fifth, a control study (exp. 4) using low-level visual stimuli also failed to show longer-term effects of sensory stimulation, despite reports of strong effects in the literature. Future studies should determine the necessary and sufficient conditions enabling robust long-term modulation of visual performance using this technique. This step is required to consider further use in fundamental research (e.g., to study neural circuits involved in selective visual processing) and potential educational or clinical applications (e.g., inhibiting socially-irrelevant aspects of face processing in autism).

## Introduction

The human brain can learn and remember complex visual patterns. These capacities are enabled by efficient neural circuitries for processing images of objects [1,2], places [3], printed words [4–6] and faces [7,8]. Neural circuits involved in such processing are further fine-tuned by learning (for review, see [9]).

Neural mechanisms of learning and memory have been studied extensively for over 40 years in rodent brain slices at the synaptic level [10]. High-frequency electrical stimulation of neural tissue increases synaptic strength (i.e., Long-Term Potentiation [LTP]), whereas low-frequency stimulation decreases it (Long-Term Depression [LTD]). Rodent *in vivo* studies [11] and also *in vitro* investigations in human neural tissue [12] have further validated the LTP/LTD mechanisms as biological models related to learning in humans by observing increased synaptic strength after learning. More recently, evidence of LTP-like effects in humans was provided by using Transcranial Magnetic Stimulation (TMS), a non-invasive method [13,14]. Importantly, interaction between real-life learning and the induced plasticity with neural stimulation has also been demonstrated [15–17]. Further, evidence suggests that it is possible to improve sensorial or motor functioning in a bottom-up way [18]. Indeed this translation of LTP/LTD principles towards non-invasive applications in humans has provoked a growing excitement in the field [19].

Interestingly, instead of modulating a whole sensorial or motor system like in TMS studies, one can even target specific neural processing through simple *sensorial* stimulation. While some studies demonstrated short term (minutes) modulatory effects of sensorial stimulation [20] others reported long-lasting effects (up to 10 days) for elementary visual processing (black and white bars stimuli) [21,22]. Moreover, longer stimulations (hours) can induce important cortical reorganization, as shown in cats with peripheral nerve stimulation [23]. Given the common properties of classical LTP/LTD *in vitro* and the results obtained with simple sensorial stimulation (i.e., frequency-dependence, long-lasting effects and glutamatergic dependency; see [24,22,25,26], both approaches seem to rely on the same synaptic mechanisms. In accordance, learning induced by natural exposure (perceptual learning) or by stimulation (LTP) present similar characteristics, such as stimulus-specificity and a minimal amount of trials/stimulation required to induce learning [25,27].

**Fig 1:**
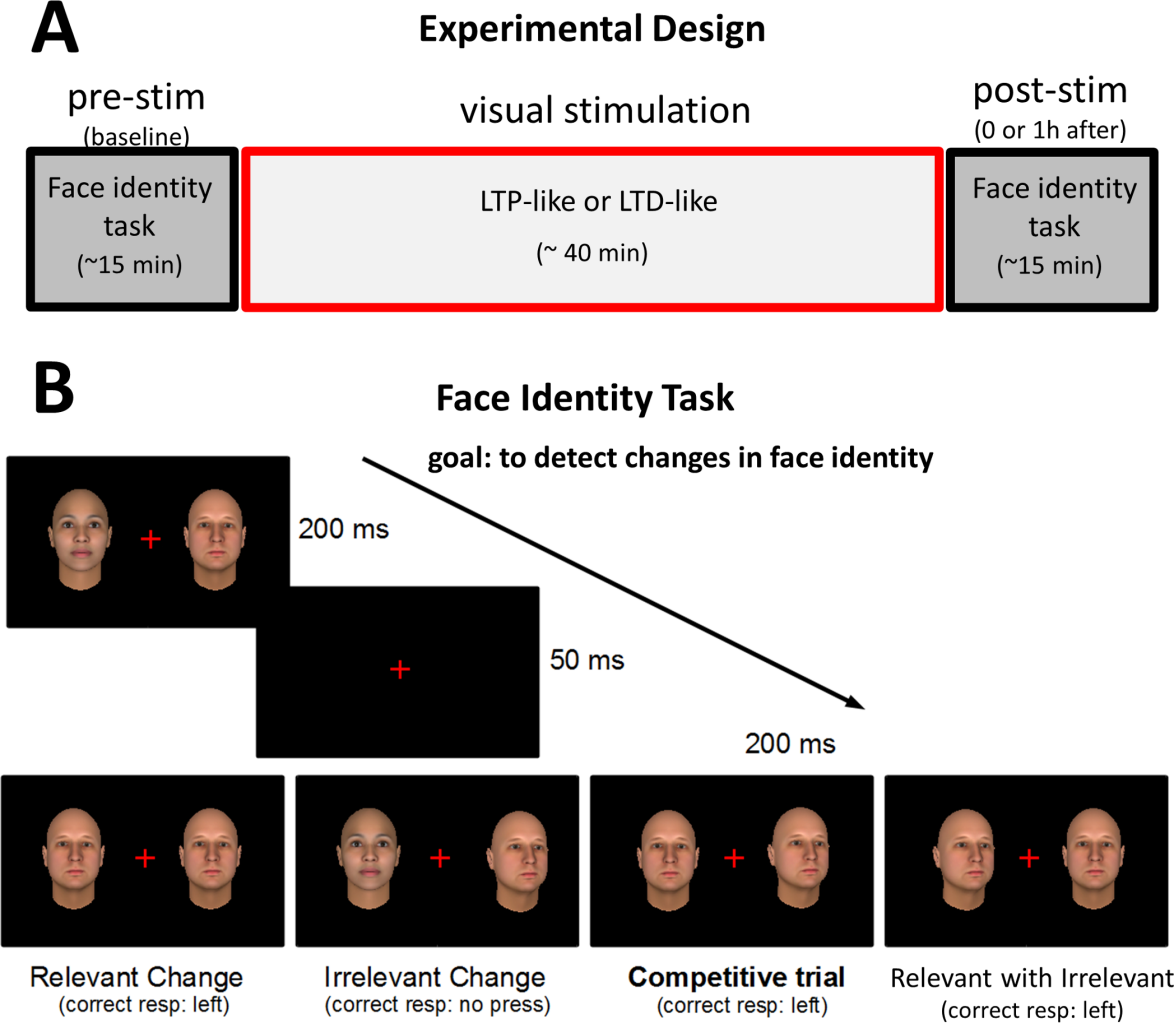
Experimental design. A) Visual performance on face processing was assessed before (pre-stimulation) and after (post-stimulation) passive visual stimulation with two different protocols (LTP-like or LTD-like). B) Face identity change detection task used in Experiments 1–3. In a sequence of screens, relevant (face identity) and/or irrelevant (face orientation) changes were randomly presented in each hemifield. Each condition (“Relevant Change”, “Irrelevant Change”, “Competitive Change” and “Relevant with Irrelevant”; see Methods) was presented an equal number of trials. One ninth of the trials presented no changes (not shown). Note the challenging nature of the competitive change trials where task-relevant *identity* changes should be detected despite the presence of a distractor (irrelevant head-orientation change) in the opposite hemifield. The correct response for these example trials is indicated below each condition.

But can we passively manipulate visual learning/unlearning in humans for complex high-level stimuli such as pictures of objects or faces? Here we explored the use of sensorial stimulation at specific frequencies either to improve or to weaken visual processing of faces. Our protocol consisted of a challenging face identity change detection task which was administered three times before and three times after the visual stimulation (see Fig 1). Visual stimulation consisted of bilateral (exp.1, 3 and 4) or unilateral (exp.2) changes of task-relevant stimuli (face identity in exp. 1, 2 and 3; simple bars in exp.4) at a high (~10Hz) or low (~1Hz) frequency rate (LTP and LTD-like stimulation, respectively; see Methods). Subjects’ performance were re-assessed immediately after (exp.1 and 2) or one hour after (exp.3 and 4) the stimulation.

## Results

### Experiment 1: Bilateral stimulation

**Fig 2:**
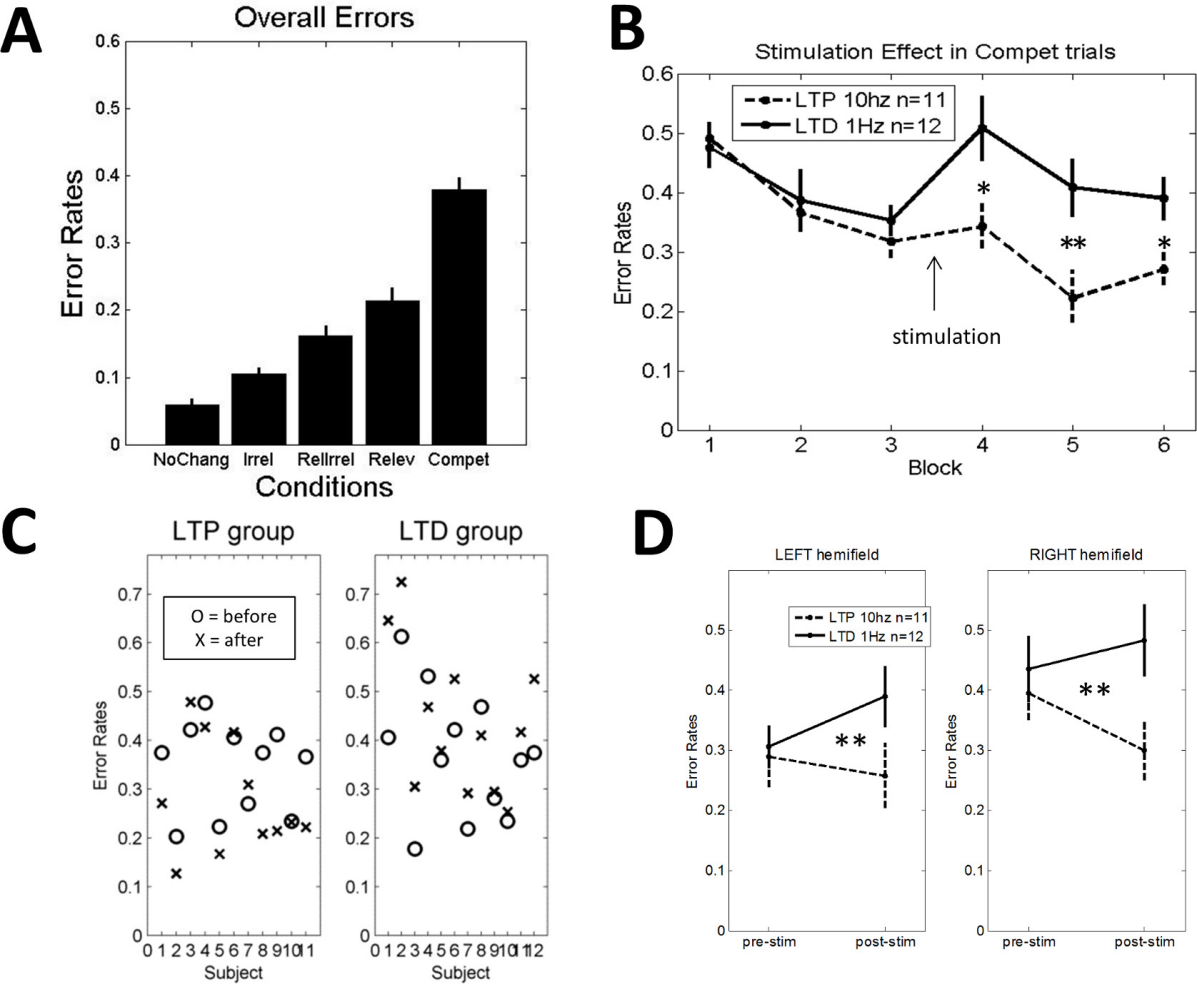
Experiment 1 (bilateral stimulation). A) Overall task performance: Error Rates for the ‘No change’, ‘Irrelevant Change’, ‘Relevant and Irrelevant Change’, ‘Relevant Change’, and ‘Competitive Trials’ conditions. Note that competitive trials (‘compet’) are the most difficult ones. Errors in this condition were used to monitor the effects of LTP- vs LTD-like stimulation. B) Performance on ‘competitive trials’ for the three pre-stimulation and three post-stimulation blocks of the LTP-like and LTD-like groups. C) Single-subject results on ‘competitive trials’ for each stimulation group. D) Error rates on ‘competitive trials’ for each hemifield. A main effect of hemifield together with a lack of significant interaction with stimulation suggest a general *left hemifield advantage* in this face processing task (see Results and S2 Fig for equivalent results in exp. 2 and 3). Pre-stim = pre-stimulation (i.e., blocks 2 and 3); post-stim = post-stimulation (i.e., blocks 4, 5 and 6) (see Results). * p< 0.05; ** p< 0.01. Error bars = +/- 1 SEM across subjects.

In the first experiment, in order to maximize potential effects of stimulation on visual performance, we administered *bilateral* stimulation and assessed the effect on face processing performance immediately after stimulation. As in previous studies [21,22], the analyses focused on the “competitive trials”, in which a relevant change at one side was accompanied by an irrelevant change at the other side. As shown in Fig 2A, “competitive trials” were the most difficult and were clearly far away from ceiling performance. Error rates (ER) in competitive trials were used as the dependent measure in a factorial Analysis of variance (ANOVA), declaring stimulation-type (LTP-like versus LTD-like) as between-subject factor and time-point (pre versus post-stimulation) as within-subject factor. In all experiments the first of the pre-stimulation blocks (baseline) was used to accustom subjects with the task and was not included in the analyses. The ANOVA revealed a significant Group x Time interaction (F(1,21) = 12.90; *p* < 0.002), indicating that the LTP-like stimulation group significantly outperformed the LTD-like group after but not before the stimulation (see Fig 2B). Post-hoc testing revealed that the groups did not differ on any of the baseline blocks (respectively p = 0.7, p = 0.7 and p = 0.39) but they did on all post-stimulation blocks, with LTP outperforming the LTD-like group (respectively p = 0.02; p < 0.01, and p = 0.02). Despite the limited number of participants in this experiment, individual results suggest that these differential modulation effects on visual performance were quite well reproducible at the single-subject level (see Fig 2C).

Note that immediately after the stimulation (block 4) there was an increase in average error rates relative to block 3, with a significant Group x Time interaction (F(1,21) = 6.31; p = 0.02), demonstrating that the LTD-like group presented a higher increase in errors (15.7%) relative to the LTP-like group (2.6%). Further, when restricting this analysis to the LTD-like group we observed a significant increase in errors (F(1,11) = 13.9; p = 0.003) while for the LTP-like group this impoverishment did not reach statistical significance (F(1,10) < 1). This effect can be due to visual fatigue or visual adaptation effects (‘face adaptation’) and will be further characterized in exp. 2 (unilateral stimulation).

Next, to verify if our task was targeting high-level visual processing we tested the presence of a *left hemifield advantage* for face processing, as reported in the literature [28]. This advantage may be due to the lateralization of the Fusiform Face Area [FFA] predominantly on the right hemisphere [7] with contralaterally biased receptive fields [29]. We thus added hemifield (Left versus Right) as a within-subject factor to the previously described ANOVA. A main effect of hemifield (F(1,21) = 8.99; p < 0.007) with significantly better performance in the left hemifield was found. Importantly, no interaction with group (F < 1) nor triple interaction (F < 1) were observed. Thus, these results indicate an equivalent effect of the stimulation on both contralateral hemispheres. Indeed, when restricting the analysis to each hemifield separately, we found a significant Group X Time interaction for the left (F(1,21) = 8.08; p = 0.0097) as well as for the right hemifield (F(1,21) = 8.48; p = 0.0083) (see Fig 2D).

A similar left hemifield advantage was found in the two other experiments involving face processing (exp.2 and 3) but not in the experiment with oriented bars (exp.4) (see S2 Fig). These results concordantly suggest that our task paradigm was targeting high-level visual processing of faces.

### Experiment 2: Unilateral stimulation

**Fig 3:**
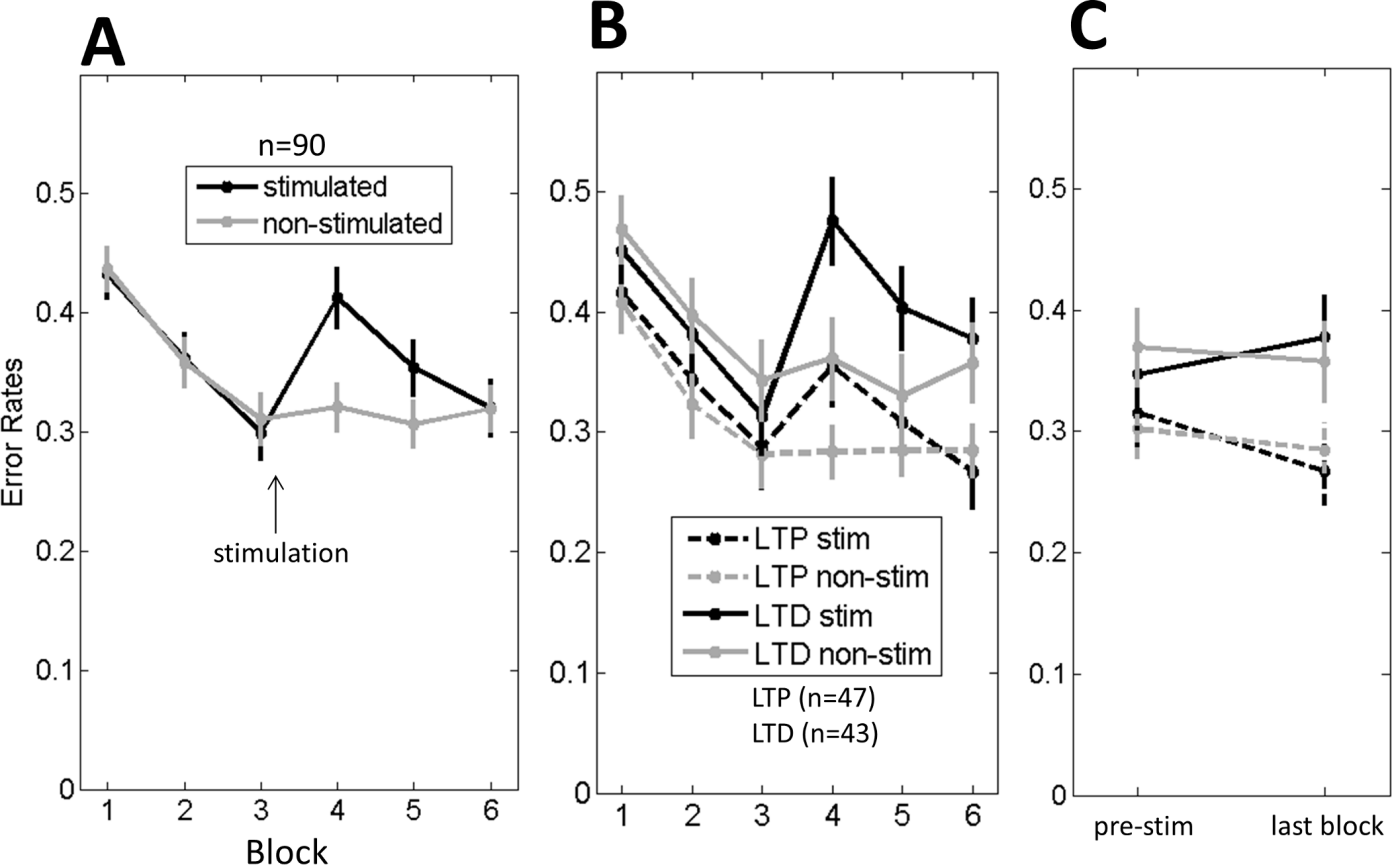
Experiment 2 (unilateral stimulation). A) Error Rates on ‘competitive trials’ for the stimulated vs non-stimulated hemifield (LTP-like and LTD-like stimulation collapsed). B) Error rates on ‘competitive trials’ for each stimulation group and hemifield: LTP stim = errors in the stimulated hemifield for the LTP-like group; LTP non-stim = errors in the non-stimulated hemifield for the LTP-like group; LTD stim = errors in the stimulated hemifield for the LTD-like group; LTD non-stim = errors in the non-stimulated hemifield for the LTD-like group. C) Stimulation effects outside the “performance-disrupted window” (i.e., block 6) compared to pre-stimulation performance (i.e. block 2 and 3). Error bars = +/- 1 SEM across subjects.

We conducted a second experiment to test hemifield-specific effects of sensory stimulation by administrating *unilateral* stimulation. All other aspects were kept constant relative to exp.1, including reassessing face processing performance *immediately after* the stimulation. First, to verify a potential general effect of the stimulation on visual performance, independently of stimulation frequency, we compared performance on the stimulated versus the non-stimulated hemifield (Fig 3A). Even more clearly than in exp. 1, we observed here a temporary drop in performance immediately after the stimulation (block 4) relative to block 3, on the stimulated side (F(1,88) = 38.6; p < 0.0001) which was not statistically significant on the non-stimulated side (F(1,88) = 3.11; p = 0.08). Note that this temporary disruption was present for both groups (see Fig 3B). As in exp.1, the LTD-like was more affected than LTP-like (Group x Time interaction: (F(1,88) = 6.7; p = 0.01). However in contrast to exp.1, here, not only the LTD-like but even the LTP-like group showed a significant increase in error rates after the stimulation (respectively F(1,42) = 35; p < 0.0001 and F(1,46) = 7.9; p = 0.007) on the stimulated side, while no effect was found on the non-stimulated side (F < 1 for both groups). Further, by comparing stimulated versus non-stimulated sides we found an interaction with Time for both LTD-like (F(1,42) = 34.7; p < 0.0001) and LTP-like groups (F(1,46) = 4.5; p = 0.04). This difference in results across experiments for the LTP-like group can possibly be linked to the competitive nature of the task (‘competitive trials’), i.e., unilateral reduction in discriminability (exp.2) inducing a more clear bias than bilateral impoverishments (exp.1).

To investigate stimulation effects outside this performance-disrupted window where visual fatigue or face adaptation could have a strong impact we analyzed error rates of the last block post-stimulation against the baseline. For the stimulated hemifield (‘stim’), we observed again the differential modulation of visual performance (Group x Time: F(1,88) = 5.9, p < 0.017), with the LTP outperforming the LTD-like group on the post-stimulation but not on the pre-stimulation blocks. This interaction was absent in the non-stimulated hemifield (‘non-stim’) (F < 1) (Fig 3C). In summary, after the disruption window we could notice again passive modulation of performance depending on the stimulation-frequency.

### Experiment 3: One hour delay post-stimulation

In the third experiment, we tested the *life-time* of these stimulation effects by introducing a one hour delay between the end of the stimulation and the post-stimulation test blocks. This delay was used for three main reasons: 1) this is the typical length of delay used in previous experiments [21,22]; 2) it may avoid the temporary disruption interference noted in exp. 2; 3) it is long enough to allow theoretical analogy with *long-term* potentiation/depression observed at the neural level. In other words, if the LTP/LTD-like sensory stimulation would effectively induce LTP/LTD at the neural level, we should notice modulatory effects on behavioral performance after one hour delay.

Furthermore, in the present experiment we also included a no-stimulation condition and an LTP-like stimulation of the task *irrelevant* change (orientation of the faces).

**Fig 4:**
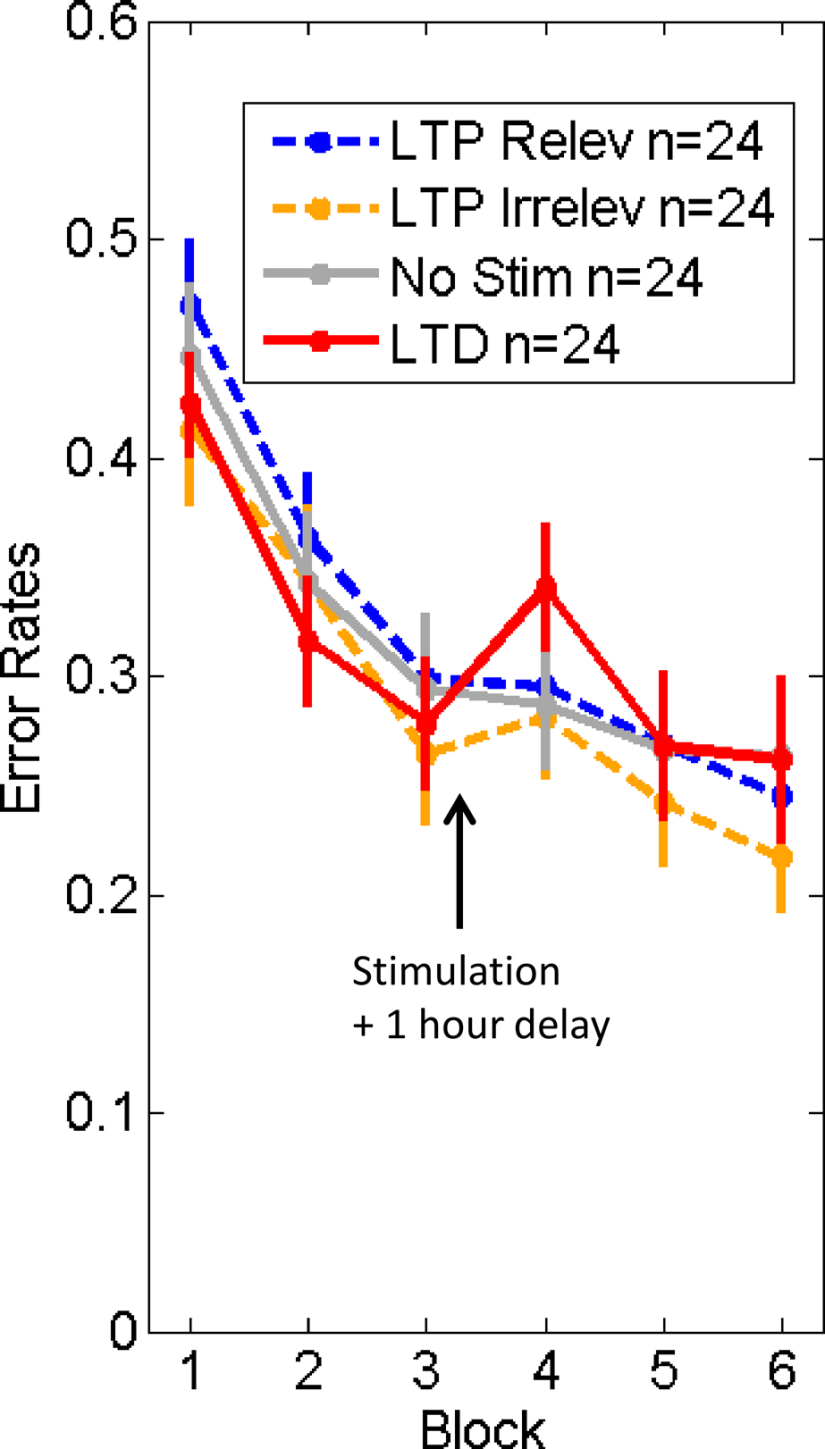
Experiment 3 (one hour delay post-stimulation). Error rates on ‘competitive trials’ for each stimulation condition: LTP relev = high-frequency relevant stimulation (face identity changes); LTP irrelev = high-frequency irrelevant stimulation (head orientation changes); No Stim = control group (without stimulation); LTD = low-frequency relevant stimulation (face identity change). See Results. Error bars = +/- 1 SEM across subjects.

Overall, we did not find a significant differential modulation of face processing performance by LTP vs LTD like stimulation. In particular there was no significant Group x Time interaction (F(3,92) = 1.85, p = 0.14), suggesting that frequency-dependent stimulation effects have vanished completely after the one hour delay period (see Fig 4). The results for the LTP-like relevant-change stimulation were strikingly similar to the no-stimulation condition, suggesting either no LTP-like effect at all or a short lasting LTP-like effect (between ~15 minutes and one hour). For the LTD-like group the pattern of results was less decisive. Despite the absence of any overall statistical effect when taking all time points into consideration, there was a suggestive drop in performance in the first testing block after LTD-like stimulation (see Fig 4). When restricting the analysis to this time point (i.e. block 4), there was a trend (F(1,93) = 2.99, p = 0.087) towards degraded performance of the LTD-like stimulation group as compared to the three other groups. This trend already disappeared from testing block 5 onwards. Thus even if the increased error rates reflected a long-lasting effect of LTD-like stimulation (present one hour later), it was transient and quickly undone after one block of performing the face processing task (see results for blocks 5 and 6). Overall, Experiment 3 does not provide convincing evidence that the LTP/LTD-like stimulation resulted in long-lasting frequency-dependent changes in behavioral performance.

### Experiment 4: Low-level stimuli and one hour delay

Finally, we performed a fourth experiment which aimed at understanding the reason for a lack of longer-term effects (> one hour), as these have previously been reported with very similar protocols but using low-level visual stimuli [21]. Essentially, we wanted to disentangle two hypotheses: 1) Are stimulation effects smaller for higher level visual processing? or 2) Is our current stimulation and test paradigm insensitive to changes in visual performance? Consequently, we tried to replicate the original findings of Beste and colleagues [21] while using our own protocol parameters. Thus, we replaced the face stimuli by black and white bars, and also added a 20Hz stimulation condition (since it was the frequency they used in the LTP-like condition), in order to verify if the stimulation frequency rate could have played a critical role. (See Methods for an explanation why we used 10 Hz for the face processing experiments).

**Fig 5:**
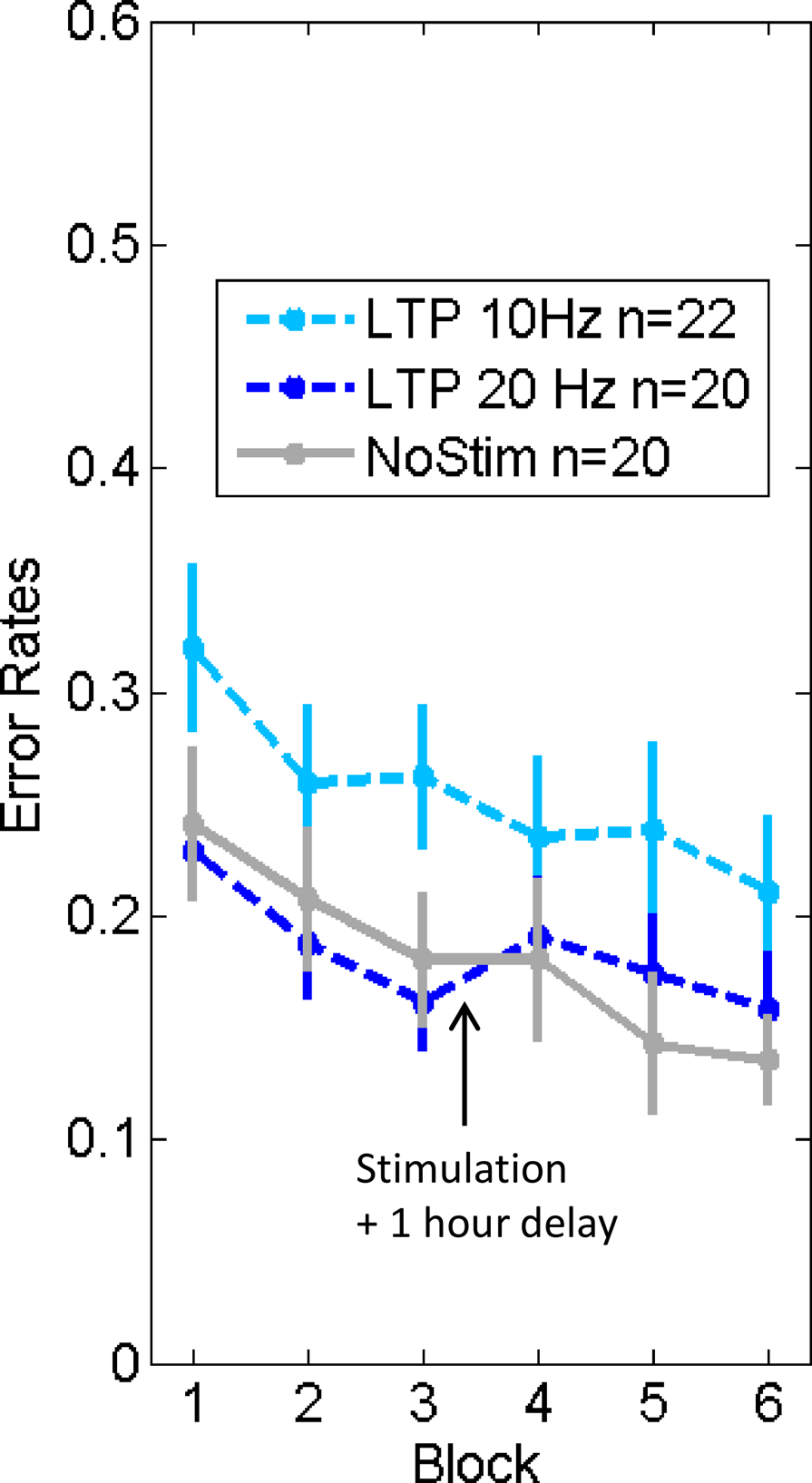
Experiment 4 (bars instead of faces; one hour delay). Error Rates on ‘competitive trials’ for each stimulation condition: LTP 10 Hz = task-relevant stimulation (luminance change) at 10Hz; LTP 20 Hz = task-relevant stimulation (luminance change) at 20Hz; NoStim = no stimulation. Arrow indicates when the stimulation was applied and followed by 1 hour delay. Error bars = +/- 1 SEM across subjects.

As illustrated in Fig 5, we could not replicate any long-term (1 hour delay) frequency-dependent stimulation effects on low-level visual processing (Group x Time: F(2,59) = 1.82, p = 0.17). It should be noted that the protocol of exp.4 still slightly differed from the one used by Beste and colleagues in the following aspects: 1) saliency level of the distractor: only one level here, while they used two levels (they found differential strength and duration of stimulation effects depending on the saliency level, and it is difficult to exactly say where saliency in our experiment falls within their tested range); 2) number of trials before and after stimulation (432 here vs 512 in Beste et al.); 3) context of conditions: we included an additional “no-change” condition in 1/9^th^ of the trials); 4) distance of stimuli to fixation cross: 2.1 degrees here vs 1.1 degree there). However, we tested 10 additional subjects with a 1.1 degree distance between stimuli and fixation cross and the performance did not change at baseline nor at post-stimulation blocks.

## Discussion

In the present work we explored the potential use of LTP/LTD-like non-invasive brain stimulation via *sensorial input* to passively manipulate visual sensitivity of humans to detect changes in highly complex stimuli (faces). We found short term (minutes) stimulation effects in the predicted direction, with the LTP-like group outperforming the LTD-like group (exp. 1). This performance modulation was hemifield-specific (exp. 2), concurring with previous reports on behavioral [21] and neural data [30]. We also noticed a temporary disruption in performance just after the stimulation, only on the stimulated side but for both types of stimulation (see Fig 3A and 3B of exp. 2). Results of both experiments suggest that these modulatory effects where present for at least 15 minutes (see block 6), a duration compatible with previously observed synaptic modulation using spike-timing dependent plasticity techniques on the visual cortex of the cat [20], and also compatible with human perceptual modulation [20] including perception of faces [31]. In complement, a third experiment (exp. 3) demonstrated the vanishing of these effects after a 1 hour delay, suggesting that the duration of effects found here ranged from 15 minutes to less than one hour. The lack of longer-term effects contrasts with previous reports using similar protocols but for low-level visual stimuli [21,30]. We thus conducted a control experiment (exp. 4) to try to disentangle between two explanatory hypotheses for our findings: A) reduced influence of sensory stimulation on higher level visual processing (e.g., effects may be restricted to low-level areas and may not propagate to higher level areas) versus B) a lack of sensitivity of our paradigm. The results of Experiment 4 shown a lack of replication of long-term stimulation effects (see Fig 5), possibly suggesting that the parameters of our test and/or stimulation protocols could have been suboptimal to demonstrate long-term effects. While we can exclude the involvement of a number of paradigm factors (see results of exp.4), the importance of the saliency of the distractors on task sensitivity, for instance, cannot be ruled out (cf. fig. 2 in [21]).

Overall, we can conclude that it is not straightforward to reveal long-term effects of LTP/LTD-like sensory stimulation, neither with high-level face stimuli nor with low-level visual stimuli. At a shorter time scale however, we could demonstrate the predicted modulation on visual performance for face discrimination as a function of stimulation protocols. We could also observe a temporary decrease in behavioral performance on the stimulated side (see Fig 3A). This can be linked to “visual fatigue” caused by the stimulation. Alternatively it can also be due to ‘visual adaptation’ to the face stimuli used during stimulation, leading to a temporary decrease in discriminability. Indeed, disrupted perception of faces can be observed after face adaptation, typically presenting an exponential decay of the effect and a duration of a few minutes [32–34], a pattern also observed in the present work. In any case, this temporary “performance disruption” observed here can possibly shed light on previous unexpected findings in the literature. In this regard, an fMRI study investigating LTP-like effects on the visual system recorded reduced instead of increased visual cortex responses relative to baseline immediately after 9Hz stimulation [35]. The “temporary disruption” effect may have masked the LTP-like effects in this time period, and the expected enhanced responses could have been obtained a few minutes later, outside the “disrupted window”. Indeed, this is exactly what was found in an EEG study using also 9Hz stimulation when subjects were retested several minutes after stimulation (up to 52 minutes) [30]. Furthermore, a recent EEG study using a parametric variation of delays after high-frequency stimulation (~9Hz) found differential modulatory effects in early components (< 250 ms) of visual evoked potentials: for 2–4 and 4–6 minute delays the evoked potentials were decreased whereas longer delays (20–22 and 120–122 minutes) revealed increased visual responses [36]. Besides the temporary disrupted effect immediately after the stimulation, we should also consider visual adaptation as an alternative explanation for the differential modulation effects found in the present experiments (exp. 1 & 2). Indeed, visual adaptation to face stimuli could have played a major role, given that the total exposure time to faces was higher for the LTD-like compared to the LTP-like group (see Methods). As a consequence, the former could have suffered more face adaptation than the later, leading to higher error rates of face discrimination after the stimulation. Note that even if total presentation time would be the same, the differential dynamics in the two conditions could give rise to differential adaptation. One stimulus presented for 5 seconds could elicit different amounts of adaptation compared to 5 stimuli of 1 second long. Visual adaptation is a complex phenomenon. Previous studies have shown that the duration of visual adaptation effects is variable (seconds to days), with mechanisms for short and long-term changes being dissociated [37] and occurring for both low [38] and high-level visual stimuli, including faces [39,40]. The underling neural plasticity has been related to changes at different levels, from cellular membrane changes [41] to synaptic [42,43] and network modulations [44]. Thus, if visual adaptation is playing a role here it is probably interfering at different levels of neural hierarchy.

Another point to be considered is the fact that interfering during the *initial* learning phase can have complex consequences, with either suppressive or facilitatory effects, as reported in the perceptual learning literature [45–47]. As can be seen in the first blocks of the task (baseline), participants were still in the middle of the steep learning phase when the stimulation was introduced. Thus, it is conceivable that the effects of the stimulation could have been confounded with this complex interaction during ongoing learning processes.

## Conclusion and Future Perspective

Brain stimulation is getting momentum in the field of cognitive neuroscience as an alternative tool for research and educational/clinical applications. Here we provide evidence of short-term effects (minutes) of passive *sensorial* stimulation on behavioral performance for high-level stimulus processing. Instead of interfering with face processing via focal brain stimulation [48] we could manipulate performance of face processing in a predictable and passive way. Future studies should determine the critical factors to induce longer-term effects (hours, days) in order to enable a robust use of this bottom-up method to selectively study neural circuits involved in a particular visual processing. Given its simplicity (indeed even recreational videos or video games could integrate the principle), this bottom-up “neuronal education” holds the potential for a wide range of applications including therapeutic interventions, as it has been proposed in the tactile sensory domain [18,49]. It may be of special interest for individuals suffering from neurodevelopmental disorders, such as autism spectrum disorder; e.g., to inhibit selective irrelevant aspects of face processing, especially given new evidence suggesting they would be more sensitive to LTP/LTD-like manipulations [50]. However, long-term effects of this sensory modulation proved difficult in our study, indicating that we still have to understand the necessary and sufficient conditions to induce such long-term effects before considering a larger use of this technique.

## Methods

### Participants

284 typically developing young adults, essentially undergraduate students from the bachelor/master programs of psychology at KU Leuven (mean age = 19.4 years, *SD* = 2.2; 235 females) participated in one of the four experiments, distributed in the following way: 26 in exp.1; 98 in exp.2; 98 in exp.3; 62 in exp.4. They were recruited via the university online experiment system for first year students, social media and publicity on the campus. Exclusion criteria were neurological disease, age > 27 years old and (uncorrected) vision problems. Subjects received student credits or 8 euros/hour in vouchers. 14 subjects were excluded because at least one of the following reasons: incomplete data, performance < 50% during stimulation or technical problem (excluded: 3 in exp.1; 8 in exp.2; 2 in exp.3; 0 in exp.4). All participants had normal or corrected-to- normal vision. The study was approved by the local ethical committee of KU Leuven University (S55601). All subjects provided written consent.

### Face identity task

To monitor face processing performance we used a face identity change detection task in exp.1, 2 and 3 (see Fig 1B). This attention competition task was modeled after the one used by Beste et al.(2011), but using faces as stimuli instead of low-level stimuli (bars). Two human faces were simultaneously presented on a computer screen, right and left from the central fixation cross. Participants had to detect changes in face identity and report the side at which they occurred by pressing the corresponding button (left or right). Irrelevant head orientation changes were also displayed to distract attention, requiring selective face processing (identity change detection) to correctly perform the task. Participants were explicitly instructed to ignore irrelevant face orientation changes. The two faces were presented for 200ms, followed by a short interval of 50ms, followed by a new screen with two faces for 200ms (see Fig 1B). In the “Relevant Change” condition, one face identity changed on one side of the screen. In the “Irrelevant Change” condition, only the orientation of one face changed. In the “Competitive trials” condition, a relevant identity change was present on one side (that should be reported) while an irrelevant orientation change was present on the other side. This latter condition was especially challenging due to the simultaneous presence of a target and a distractor change. In the “Relevant with Irrelevant” condition both kinds of change occured at the same side. In the “No Change” condition (not depicted in Fig 1), neither of the two faces changed. All conditions were equivalently present on the left and right hemifields and with an equal amount of trials, with exception of the “No Change” condition that only represented 1/9^th^ of the trials. Three blocks of 144 trials each (~ 5 minutes duration each) were administered before stimulation (pre-stimulation) and three blocks after stimulation (post-stimulation). The first trial of each block was not analyzed to avoid surprise effects of the first stimulus. Conditions were explicitly shown to the subjects prior to the task. The same procedures were applied in exp. 4 but faces were replaced by black and white bars which changed in terms of luminance (relevant change) and orientation (irrelevant change) (cf. Beste et al., 2011). Correctness of responses was favored instead of speediness. In all four experiments, Response Time (RT) measures were not sensitive to the stimulation manipulation.

### Stimulation protocols

After obtaining baseline (pre-stimulation) measures on the face identity task (three blocks), participants underwent one of the two visual stimulation protocols (LTP-like versus LTD-like) for 40 minutes (10 mini-blocks of 4 minutes). During stimulation, for all four experiments, participants performed an orthogonal task, by detecting subtle and fast shifts of the central fixation cross to the left or right side of the screen. This task was used to guarantee that participants were fixating correctly and thus receiving the stimulation at the same location on their retina but also to insure they were not paying attention to the peripheral flickering of stimuli. Note that it was an engaging task since we used subtle fixation shifts. In experiment 1, 3 and 4, subjects received *bilateral* stimulation. In experiment 2, they received *unilateral* stimulation on only one hemifield (left or right) during the entire stimulation protocol while the non-stimulated side presented no-stimulus or a static one (counterbalanced across participants).

The stimulation consisted of face switches between the same pair of stimuli used in the face identity task (only two different faces were used both in stimulation and task; see Fig 1B) at different rates. The LTP-like group received stimulus changes at ~10 cycles/second (10.6 with 85Hz monitor for exp.1 & 2; 10.0 with 100Hz monitor for exp.3 & 4) during 5 seconds-on and 5 seconds off. The LTD-like group received stimulation at approximately 1.0 cycle/second, presented continuously. These protocol parameters were chosen given the previously reported effective induction of LTP and LTD-like effects on visual performance [21]. Note however that for high-frequency stimulation we preferred to use 10Hz instead of 20Hz for two main raisons: 1) high-level visual areas process visual information in lower frequency ranges than low-level visual areas [51,52] and 2) to avoid perceptual fusion between the two face identities when increasing the flickering rate above 10Hz. For each 10 seconds period, the head position (frontal or lateral view) was fixed and only faces identity changed. We used both head positions during the stimulation, interleaved across 10 seconds periods, for all stimulation groups.

Two additional groups were included in exp.3: “LTP-irrelevant” where the stimulation targeted the *irrelevant* distraction change (head position), i.e., flickering of the same face in the two head positions; and “No Stim” group, without stimulation but performing the same orthogonal task on the fixation-cross.

In exp.4, the stimulation consisted of bilateral luminance changes (black and white) of the bars. As in previous experiments, we used both orientations of the bars, interleaved across 10 seconds periods.

### Apparatus

The visual stimuli were presented with 85Hz (exp.1 and 2) or 100 Hz (exp.3 and 4) CRT monitors, 15,6”large, placed at approximately 85cm from the participant in a dark room. Chin fixation was applied in order to keep the distance between the eyes and the screen constant and to guarantee that stimuli arrived at the same retinal location for both the task and stimulation. Each face stimulus subtended 7.3 x 4.6 degrees of visual angle (vertically and horizontally respectively). Stimuli were presented bilaterally and were equidistant from the central fixation cross of approximately 2.1 degrees. Responses were recorded with a standard keyboard. Psychtoolbox [53,54] a free toolbox of Matlab (The MathWorks Inc., Natick, MA, 2000) was used to deliver stimuli for the task and stimulation. Statistics were performed in R software (www.r-project.org). Face stimuli were created with FaceGen software (http://facegen.com).

## Supporting Information

S1 Fig Error rates (ER) and Response Times (RT) for experiments 1–4. See Fig 2A for ER of Experiment 1. Conditions: NoChang = ‘No change’; Irrel = ‘Irrelevant Change’; RelIrrel = ‘Relevant and Irrelevant Change’; Relev = ‘Relevant Change’; Compet = ‘Competitive Trials’. Error bars = +/- 1 SEM across subjects.
(TIFF)

S2 Fig Main effects of Hemifield. In all experiments using faces (Exp. 1, 2 & 3) a significant left hemifield advantage was noticed (p < 0.01 for all), a reported finding in face processing studies, probably linked to the location of Face Fusiform Area on the contralateral hemisphere. In contrast, for Exp.4 using low-level stimuli (black and white bars), no hemifield advantage was noticed. Error bars = +/- 1 SEM across subjects.
(TIFF)
