# Supplementary figures and images for: Exploring the Use of Sensorial LTP/LTD-Like Stimulation to Modulate Human Performance for Complex Visual Stimuli
Source: PLoS One. 2016 Jun 24;11(6):e0158312. doi: 10.1371/journal.pone.0158312 (PMC4920386; doi:10.1371/journal.pone.0158312)

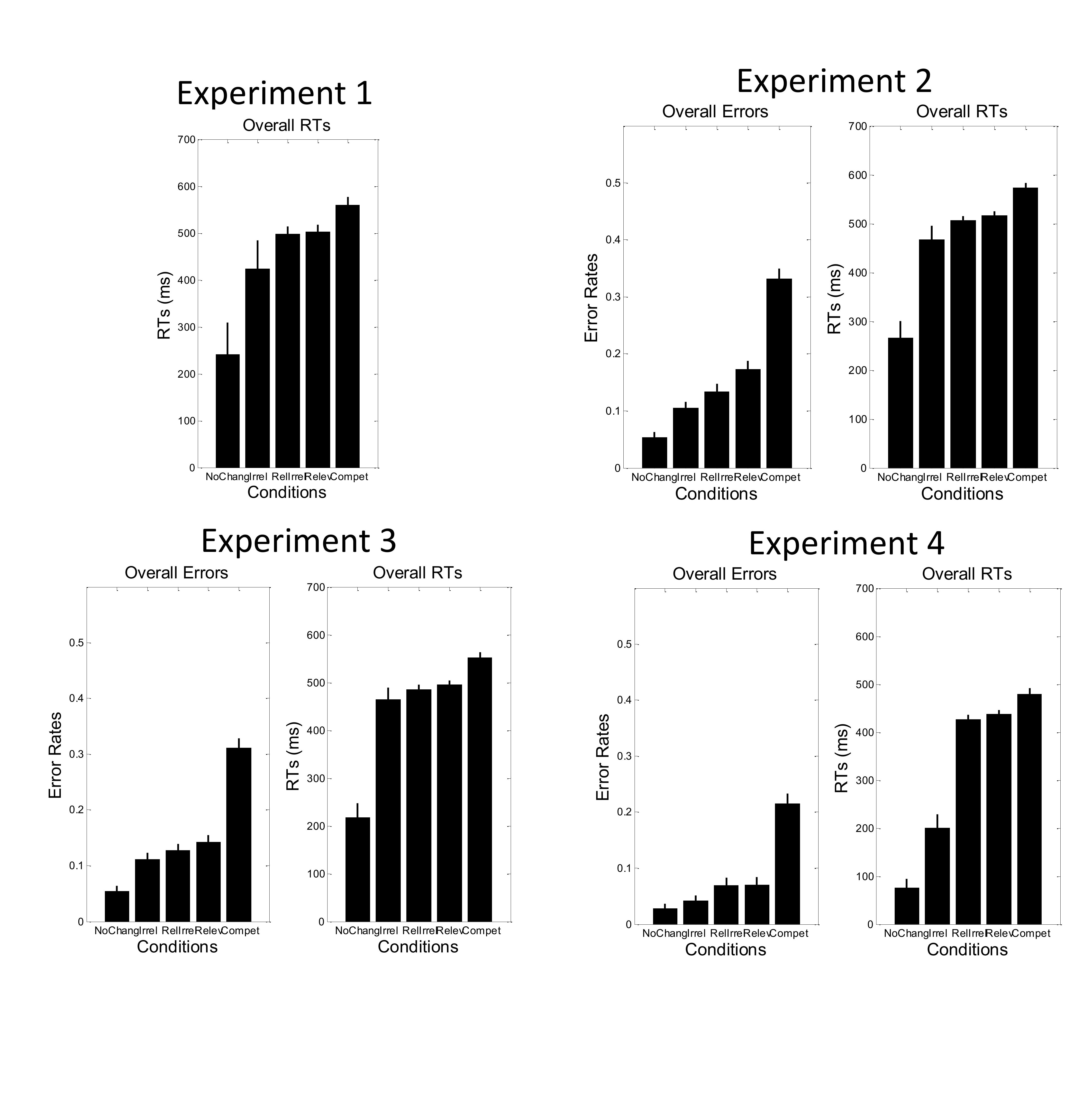

Supplement: S1 Fig — See Fig 2A for ER of Experiment 1. Conditions: NoChang = ‘No change’; Irrel = ‘Irrelevant Change’; RelIrrel = ‘Relevant and Irrelevant Change’; Relev = ‘Relevant Change’; Compet = ‘Competitive Trials’. Error bars = +/- 1 SEM across subjects. (TIFF) [file pone.0158312.s001.tiff]

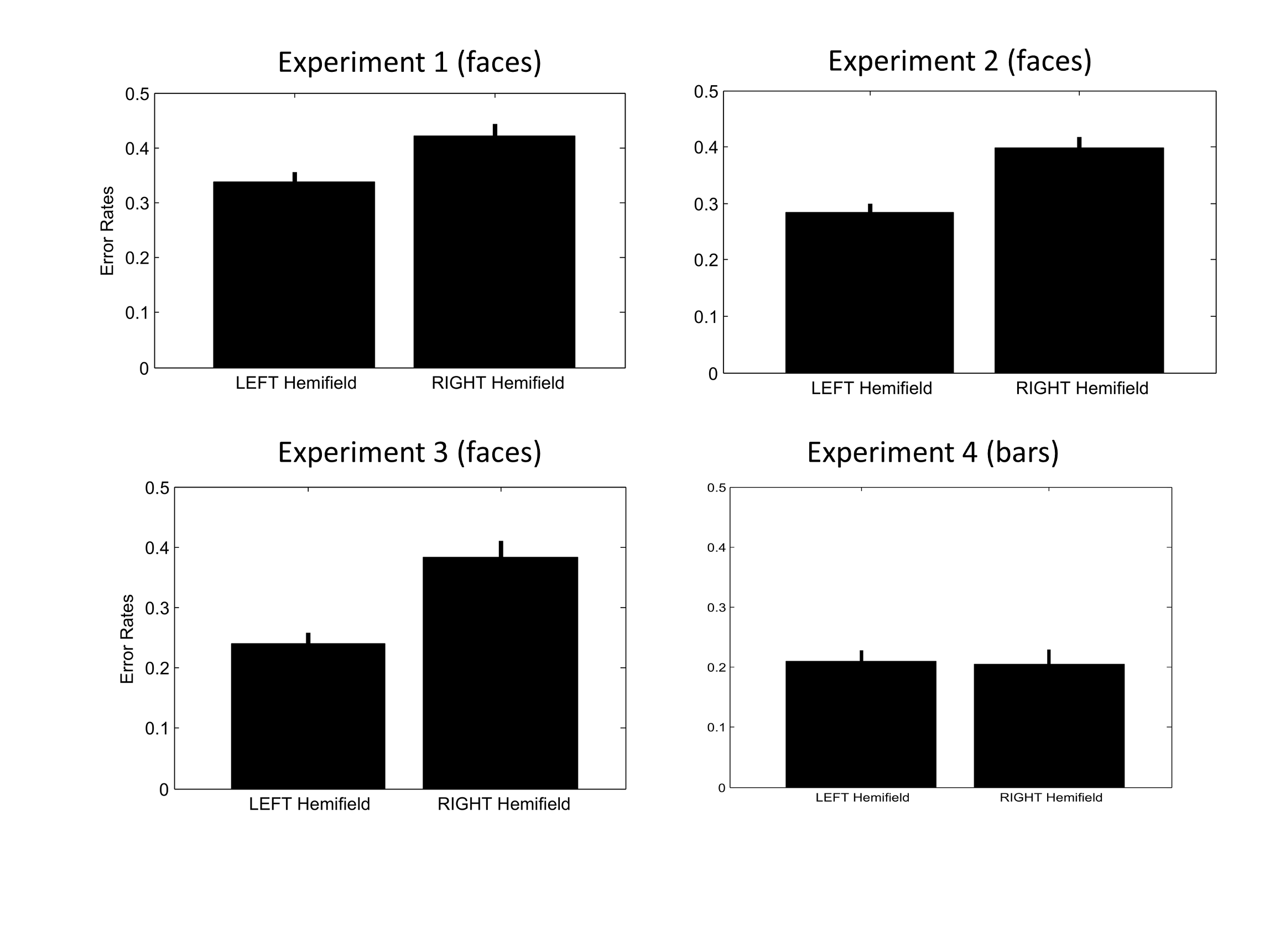

Supplement: S2 Fig — In all experiments using faces (Exp. 1, 2 & 3) a significant left hemifield advantage was noticed (p < 0.01 for all), a reported finding in face processing studies, probably linked to the location of Face Fusiform Area on the contralateral hemisphere. In contrast, for Exp.4 using low-level stimuli (black and white bars), no hemifield advantage was noticed. Error bars = +/- 1 SEM across subjects. (TIFF) [file pone.0158312.s002.tiff]
